# A System for Analog Control of Cell Culture Dynamics to Reveal Capabilities of Signaling Networks

**DOI:** 10.1016/j.isci.2019.08.010

**Published:** 2019-08-08

**Authors:** Chaitanya S. Mokashi, David L. Schipper, Mohammad A. Qasaimeh, Robin E.C. Lee

**Affiliations:** 1 Department of Computational and Systems Biology, School of Medicine, University of Pittsburgh, Pittsburgh, PA 15213, USA; 2 Division of Engineering, New York University Abu Dhabi, Abu Dhabi, UAE; 3 Mechanical and Aerospace Engineering, Tandon School of Engineering, New York University, Brooklyn 11201, USA

**Keywords:** Immunology, Mathematical Biosciences, Complex Systems, Biological Science Instrumentation

## Abstract

Cellular microenvironments are dynamic. When exposed to extracellular cues, such as changing concentrations of inflammatory cytokines, cells activate signaling networks that mediate fate decisions. Exploring responses broadly to time-varying microenvironments is essential to understand the information transmission capabilities of signaling networks and how dynamic milieus influence cell fate decisions. Here, we present a gravity-driven cell culture and demonstrate that the system accurately produces user-defined concentration profiles for one or more dynamic stimuli. As proof of principle, we monitor nuclear factor-κB activation in single cells exposed to dynamic cytokine stimulation and reveal context-dependent sensitivity and uncharacterized single-cell response classes distinct from persistent stimulation. Using computational modeling, we find that cell-to-cell variability in feedback rates within the signaling network contributes to different response classes. Models are validated using inhibitors to predictably modulate response classes in live cells exposed to dynamic stimuli. These hidden capabilities, uncovered through dynamic stimulation, provide opportunities to discover and manipulate signaling mechanisms.

## Highlights

• Automated and accessible analog control of dynamic cell culture microenvironments
• Gravity-driven flow enables long-term modulation for multiple time-varying stimuli
• Atypical patterns of NF-κB activation are revealed by dynamic stimulation with TNF
• Protein translation rate is a source of variability between NF-κB response classes

## Graphical Abstract

**Figure:**
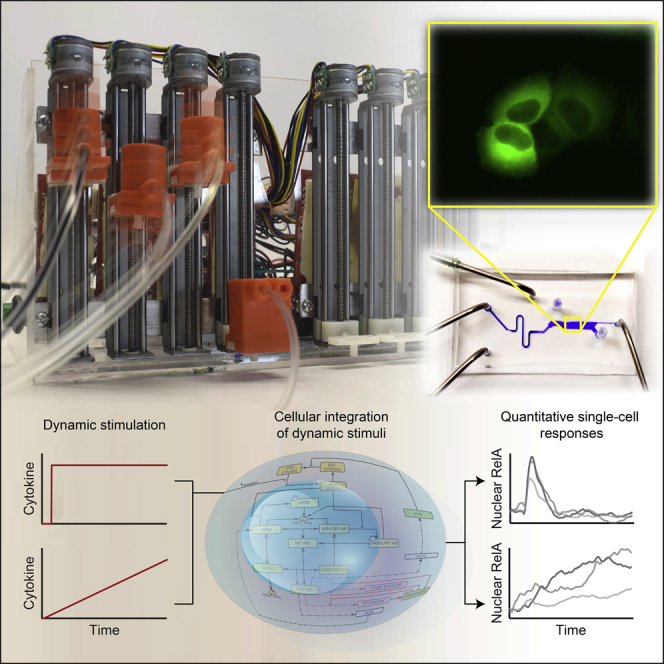

## Introduction

The microenvironment of a cell is multifarious, with a constantly changing composition of extracellular molecules. When cells are exposed to extracellular cues, such as changes in the concentration of inflammatory cytokines or drugs, they activate dynamic signal transduction pathways within the cell that govern pivotal cell fate decisions (Albeck et al., 2013, Lee et al., 2016, Purvis et al., 2012, Purvis and Lahav, 2013, Ryu et al., 2016, Spencer et al., 2009). Although deregulation of these pathways contributes to human disease, most experiments characterize cells exposed to constant stimulation, which contrasts the transient and time-varying properties of cues *in vivo*.

Biomedical micro-electro-mechanical systems such as microfluidic devices broadly enable studies of cell behavior in precisely controlled microenvironments (Mehling and Tay, 2014, Whitesides, 2006). Microfluidic flow systems that operate by switching between multiple inlets, each with discrete concentrations or different stimuli, can generate time-varying changes of medium composition in a cell culture (Gomez-Sjoberg et al., 2007, He et al., 2015, Kim et al., 2012, Lee et al., 2008, Lee et al., 2009, Piehler et al., 2017). However, owing to their digital design, switch-based approaches often have limited operating ranges (usually under 10-fold dynamic range of concentration dilutions) and rely on specialized expertise and equipment to make and use. Other approaches that dilute fluids between reservoir pairs to alter the composition of a cell culture medium (Bennett et al., 2008, Hersen et al., 2008) require high-precision flow control systems that are not available in most biology laboratories. Because of these challenges, signal transduction networks are rarely investigated in the context of dynamic microenvironments.

We set out to address these challenges by first developing a minimal dynamic stimulation system that provides time-varying control over cell culture composition and then establish proof of concept by using the system with live-cell imaging experiments. The modular dynamic stimulation system consists of a gravity pump controller, which can be built from commonly available low-cost parts, to coordinate gravity-driven flow (Lee et al., 2016) and laminar fluid streams in a cell culture device (Kuczenski et al., 2007, Ryu et al., 2018, Takayama et al., 2003). Cell culture devices under control of the gravity pump are assembled from 3D-printed molds and can be interchanged to provide different functions. By automating flow rates in the cell culture device, the system provides independent control over time-varying concentrations for multiple stimuli over a broad dynamic range. To demonstrate use of the system, we compare activation of the nuclear factor (NF)-κB signaling pathway in live cells exposed to tumor necrosis factor (TNF) either as a step-up to a continuous concentration or as a slow ramp of increasing concentration. Although the characteristic response of the NF-κB pathway is adaptive, we find alternative patterns of pathway activation when cells are exposed to a TNF ramp. We also find that pathway activation is greater in response to ramp stimuli, even though cells are exposed to significantly less TNF over the duration of the experiment. Using computational models and validation experiments, we show that cell-to-cell variability for negative feedback within the NF-κB signaling network determines the pattern of pathway activation in cells exposed to step and ramp stimuli. Our results demonstrate that dynamic stimulation can be achieved under gravity-controlled flow with the system developed here and can be used to reveal hidden capabilities of signal transduction networks.

## Results

### Development of a Dynamic Stimulation System for Live-Cell Imaging

**Figure 1:**
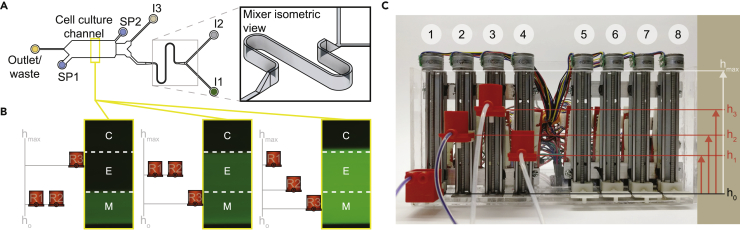
Gravity Pump and Cell Culture Chamber for Dynamic Stimulation (A) Top view of the dynamic stimulation device with three inlets (I1, I2, I3), two cell-seeding ports (SP1 and SP2), and an outlet (see also Data S1). Inlets I1 and I2 are followed by a raised mixer (inset) that dilutes stimulus to desired concentrations according to flow rates from I1 and I2. I3 controls the laminar interface position (LP) of the experimental band in the cell culture channel. Cells are seeded from SP2 into the cell culture channel and observed by time-lapse imaging. See also Figure S1. (B) Flow rates through the inlets (I1, I2 and I3) are controlled by hydrostatic pressure differences between corresponding reservoirs (R1, R2, and R3) and the outlet. In the default position (left), R3 is positioned higher such that the stimulus from the mixer is confined only to the mixer band (“M”). During experiment (center and right), R1 and R2 are positioned higher to move the LP over the experimental band (“E”). Volume fraction (Xc) of stimulus in the experimental band is determined by the relative positions of R1 and R2. Control band (“C”) is not exposed to stimulus during the experiment. (C) The “gravity pump” consists of eight vertically mounted stepper motors with screw-nut platforms and an Arduino microcontroller to control platform heights; 3D printed reservoirs (see also Data S2) on platforms 1–4 are connected to corresponding inlets via tubing. Differences between inlet (h1, h2, and h3) and outlet (h0) reservoir heights determine the hydrostatic driving pressure at each inlet. See also Video S1.

We designed a microfluidic dynamic stimulation device with architectural features in the order of tens of microns or larger to be compatible with resolutions of common stereolithography 3D printers (Figure S1). When used as a negative relief for polydimethylsiloxane (PDMS) device fabrication, 3D-printed molds circumvent the need for specialized photolithography and microfabrication facilities (Ho et al., 2015) and allow for high-aspect-ratio integrated devices that are otherwise very challenging to fabricate (Brimmo et al., 2018). The device design consists of three control inlets (I1, I2, and I3), cell-seeding ports (SP1 and SP2), a mixer region, a cell culture channel, and an outlet (Figure 1A). Continuous flow is established when fluid-containing reservoirs (R1, R2, and R3) are attached via tubing to the device and at least one of the inlet reservoirs is positioned above the outlet. Flow rates from each of R1, R2, and R3 are controlled by moving their vertical positions with respect to each other and the outlet.

Taking advantage of the variable microchannel heights that can be produced by 3D printing, we designed a raised micro-mixing channel downstream of I1 and I2 and before the cell culture channel (Figure 1A, inset), which would permit efficient diffusive mixing between the two streams. Downstream of the mixer, we found that fluorescence from Alexa 488-conjugated BSA added to cell culture medium in R1 was diluted by non-fluorescent “Medium only” from R2 with a homogeneous spatial distribution that indicates efficient mixing (Figures 1B and S2). Differences between combined driving pressures from reservoirs R1 and R2 and that from reservoir R3 are used to move the laminar interface position (LP) of the mixer stream within the cell culture channel. The cell culture channel can be divided into distinct parallel bands of user-defined width along the direction of flow (Figure 1B). In the default reservoir operating position (Figure 1B, left), a narrow band of the cell culture channel is continuously exposed to the output from the mixer (M) and the rest of the channel is exposed to “Medium only.” During the experiment, the LP of the mixer stream is moved to include the experimental band (E) of the channel, leaving a negative “Control” (C) that is exposed to “Medium only” throughout the experiment (Figure 1B, middle and right). During the experiment, the volume fraction (Xc), which establishes the concentration of the solute flowing from R1 over the “E” and “M” bands of the device, is determined by the relative positions of R1 and R2.

To reliably control flow rates from R1, R2, and R3, we developed a gravity pump consisting of an assembly of eight vertically oriented stepper motors attached to a chassis (Figure 1C). Each stepper motor is individually addressed by an Arduino microcontroller and rapidly controls the height of reservoir platforms (Figure 1C and Video S1). When R1, R2, R3, in addition to an outlet reservoir are attached to the platforms and connected to the dynamic stimulation device, relative changes between heights of inlet reservoirs can be used to alter their respective flow rates into the cell culture device.

Video S1. Gravity Pump Operation, Related to Figure 1 The “gravity pump” consists of eight vertically mounted stepper motors with screw-nut platforms and an Arduino microcontroller to control platform heights. When fluid reservoirs are attached to the platforms, differences between inlet and outlet reservoir heights determine the hydrostatic driving pressure at each inlet.

### Gravity-Driven Control and Modularity of the Dynamic Stimulation System

A physical model of the system was calibrated to experiments and used to determine Xc and LP in the device for combinations of reservoir positions (Figure 1C; heights h1, h2, and h3). Using Alexa 488-conjugated BSA in reservoir R1 and fluorescent tracer beads suspended in medium in all inlet reservoirs, we found that Xc and the volume flow rate (Qc) in the cell culture region can be independently varied (Figure S2). Qc was set (*Qc* = 5×10^−11^ *m*^3^/*s*) such that shear stress on cells is constant (<0.05 Pa, based on simulations) throughout subsequent experiments. Cells grown in the dynamic stimulation system therefore experience shear forces that are orders of magnitude smaller than endothelial cells in vasculature (Hsieh et al., 2014) and do not activate shear stress response signaling via mechanotransduction (Lee et al., 2016, Nagel et al., 1999).

**Figure 2:**
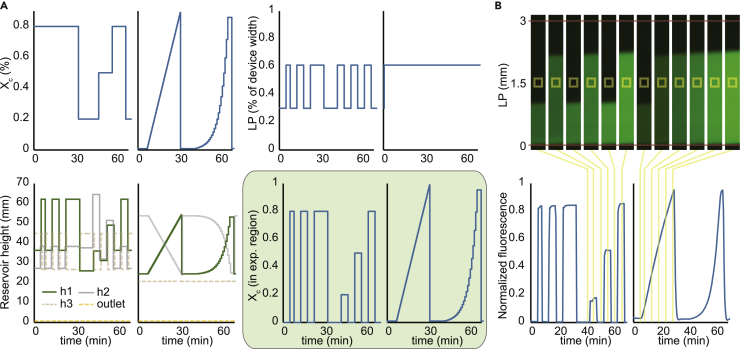
Automated Control of the Dynamic Stimulation System (A) Experiments are user defined by temporal profiles of volume fraction of cytokine (Xc; top left), laminar position (LP; top right), and flow rate (Qc; set to a constant value of *Qc* = 5 × 10^−11^ *m*^3^/*s* throughout each experiment). Using a physical model, the user-defined profiles for Xc, LP, and Qc are converted to time-varying reservoir heights (bottom left). Temporal profiles for reservoir heights are loaded on the gravity pump and run during the experiment. Green panel (bottom right) shows the predicted time-varying profile for Xc in the “E” band of the dynamic stimulation device. (B) Fluorescence intensity of Alexa 448-conjugated BSA (top) measured across the cell culture channel (yellow box in Figure 1A). Observed fluorescence in the “E” band matches predicted Xc within 5% error (bottom). See also Figure S2 and Video S2.

In principle, the system is fully analog and capable of producing arbitrarily complex patterns of stimulation in the “E” band of the cell culture channel. By adding Alexa 488-conjugated BSA in reservoir R1 as a surrogate for dilution of a stimulus, we set out to demonstrate basic capabilities of the system. In the first experiments (left side graphs of Figures 2A and 2B; see also Video S2) we used the system to generate sharp laminar pulses of varied duration or concentration. By defining the time-varying functions of Xc and LP at a fixed flow rate, the calibrated model generated time-varying profiles for h1, h2, and h3 (Figure 2A, bottom left). The predicted experimental profile (Figure 2A, green panel) agreed strongly with the fluorescence time course measured in the device (Figure 2B). Next, we used the system to generate linear and exponential ramps in concentration with a fixed Qc and LP. Although in extreme positions for h1 and h2 we occasionally observed cross-flow between the channels connecting I1 and I2 to the mixer, the system robustly produced linear and exponential ramps between Xc = 0.05 and Xc = 1.0 (Figures 2A and 2B, right side), a 20-fold dynamic range. Our results show that hydrostatic pressures achieved with the gravity pump are sufficient to precisely control the dynamics of medium composition in the cell culture channel.

Video S2. Dynamic Stimulation System Used to Dilute a Visible Dye, Related to Figure 2 Time-lapse image of dynamic stimulation system used with a visible dye. User-defined Xc and LP time courses are displayed on the left.

**Figure 3:**
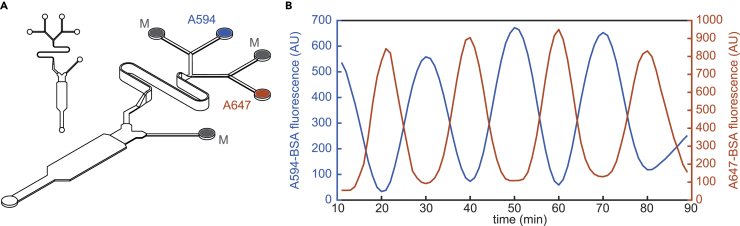
Modularity of the Dynamic Stimulation System (A) A variant device with four inlets to the mixer for simultaneous control of multiple distinct stimuli. Each inlet is connected to reservoirs containing growth medium or different stimuli. (B) Example experiment using reservoirs with Alexa 594- and Alexa 647-conjugated BSA (A594 or A647, respectively, in A) connected to two of the mixer inlets. The other inlets are connected to reservoirs with Medium only (M). Resulting fluorescence measured at the same point in the “E” band of the cell culture device shows that out-of-phase oscillations can be achieved.

Modifications to the system or the architecture of the cell culture device can provide additional functionality. For example, the stable range of dilutions can be further increased by incorporating inexpensive capillary resistors (Mavrogiannis et al., 2016) to precisely limit flow in the tubing upstream of the device and prevent cross-flow at even lower Xc values. Similarly, altering architectural properties of the device by adding additional inlet channels to the mixer (Figure 3A) broadens the stable operating range of Xc multiplicatively by over 20-fold per inlet. Theoretically, a mixer with three inlets should be stable over a 400-fold dynamic range (0.0025 ≤ Xc ≤ 1.0), and a mixer with four inlets, over 8,000-fold. Alternatively, by taking advantage of several inlets to the mixer, independent control of time-varying profiles for multiple stimuli can be achieved in a single device (Figures 3A and 3B). For a given experiment, the cell culture device attached to the gravity pump can be selected to provide stable control over a specific range of operating conditions or to address biological questions with increased complexity.

### Ramp Stimulation Reveals Distinct Modes of NF-κB Pathway Activation

The acute inflammatory response to injury and infectious agents is dynamic. Time-varying expression of pro-inflammatory and anti-inflammatory cytokines from infiltrating leukocytes, macrophages, and T cells, in addition to tissue-resident cells, determines whether inflammatory conditions are resolved and can lead to disease or sepsis when deregulated (Fullerton and Gilroy, 2016, Kumar et al., 2004, Medzhitov, 2008). At the cellular level, inflammatory cytokines such as TNF induce translocation of the NF-κB transcription factor from the cytoplasm into the nucleus, encoding a dynamic master signal for transcription of gene families (Lee et al., 2014, Lee et al., 2016, Zambrano et al., 2016, Zhang et al., 2017). When in the nucleus, NF-κB regulates several pathways of negative feedback that promote its nuclear export and retention in the cytoplasm (Ashall et al., 2009, Hoffmann et al., 2002). Consequently, during persistent stimulation with high concentrations of TNF, nuclear NF-κB often adapts back to pre-stimulus subcellular distributions.

**Figure 4:**
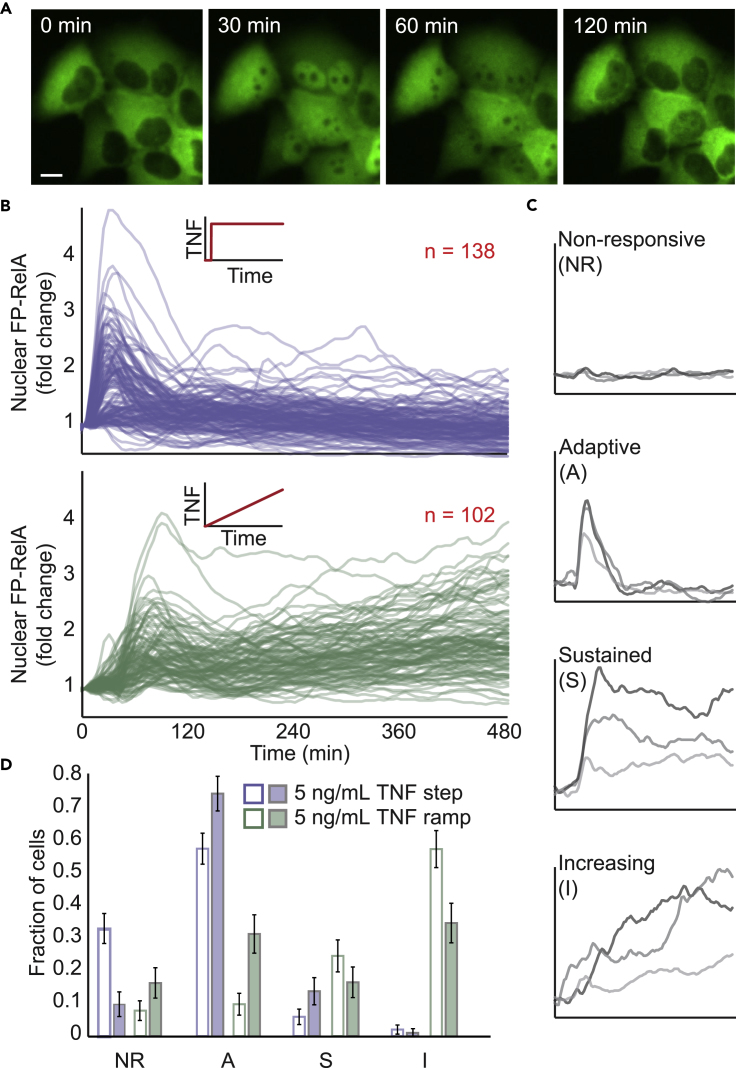
NF-κB Pathway Responses to Step and Ramp Stimulation in Single Cells (A) Time-lapse images of FP-RelA-expressing HeLa cells exposed to TNF stimulation as a step-up to continuous 5 ng/mL at the 0-min time point. Scale bar, 10 μm. (B) Time courses of nuclear FP-RelA fold change measured in single cells exposed to TNF stimulation as a 5-ng/mL TNF step (top, see also Video S3) or a concentration ramp from 0 to 5 ng/mL (bottom) over an 8-h period for a representative experiment. Raw unprocessed time courses are shown in Figure S3. Inset numbers indicate the total number of cells per condition. (C) Time courses in (A) are classified into four cellular response modes: Non-responsive, Adaptive, Sustained, and Increasing. Representative single-cell responses are depicted for each. See also Figure S3. (D) Fraction of single cells in each response mode for step and ramp stimulation show statistically significant differences in their distributions (p value < 0.00001; Pearson's chi-squared test). Independent biological replicates are shown as open and closed bars (Replicate 1: 138 cells step and 102 cells ramp; Replicate 2: 87 cells step and 80 cells ramp). Error bars represent standard deviation of 5,000 bootstrap samples. See also Figures S3–S7.

To demonstrate proof-of-concept for the dynamic stimulation system with living cells, we asked whether cells exposed to dynamic stimulation with TNF will alter the characteristic NF-κB response. HeLa cells that express a fluorescent protein fused to the NF-κB RelA subunit (FP-RelA) were seeded into the device and observed via time-lapse imaging within 3 days. The nuclear fluorescence of FP-RelA was measured in single cells exposed to either a step-like change in TNF concentration from 0 to 5 ng/mL (Video S3) or a slow ramp in concentration from 0 to 5 ng/mL over an 8-h period (Figures 4A, 4B, and S3). Consistent with our previous observations in HeLa cells (Lee et al., 2016), flow conditions did not have appreciable effects on nuclear NF-κB dynamics (Figure S4). Bioactivity of TNF also remained stable throughout the duration of experiments (Figure S5).

Video S3. Nuclear FP-RelA Mobilization in Response to a TNF Step, Related to Figure 4 Time-lapse image of nuclear FP-RelA in single HeLa cells exposed to TNF stimulation as a 5-ng/mL TNF step.

Previously we characterized adaptive and non-responsive classes of nuclear NF-κB dynamics in cells exposed to TNF as a step or a pulse (Zhang et al., 2017). For ramp-stimulated cells we observed qualitatively different single-cell responses where NF-κB remained in the nucleus during stimulus and also where NF-κB slowly accumulated in the nucleus over time (Figures 4B and 4C). We refer to these, respectively, as sustained and increasing response classes. Based on quantitative descriptors for time-varying properties of nuclear FP-RelA (Figures S3 and S6; see also Transparent Methods), the response mode for each cell trajectory was classified as either Non-responsive (NR), Adaptive (A), Sustained (S), or Increasing (I) for step and ramp stimulation conditions (Figure 4C). Although additional response classes may exist, the four response modes were robustly detected using an automated classifier (Figures S3C–S3E). Response classes found using the automated classifier were also consistent with those identified by manual inspection of data. For cells exposed to step stimulation, the clear majority showed “Adaptive” behavior followed by a subpopulation of “Non-responsive” cells and only smaller fractions of cells in the other modes. By contrast, most cells exposed to ramp stimulation showed a predominantly “Increasing” response pattern (Figure 4D). Enrichment of the “Increasing” followed by “Sustained” cellular response modes in response to a TNF ramp was at the expense of adaptive responses. “Increasing” responses were also consistently enriched in experiments wherein cells were exposed to a more rapid ramp to 5 ng/mL over a 5-h period (Figure S7). In both experiments, distributions of response modes when comparing step and ramp stimulation were statistically significant (Pearson's chi-squared test). These data demonstrate that modes of pathway activation are modulated by dynamic TNF stimulation, suggesting that the classical “Adaptive” response may be a consequence of step-like stimulation and not a defining characteristic of the signaling network.

### Aggregate Responses of Cells to Ramps Are Greater Than Step Stimulation

The “area under the fold change curve” (AUC) is a descriptor of nuclear FP-RelA dynamics that represents the accumulated response of a single cell to cytokine stimulation (Zhang et al., 2017). Comparisons of nuclear NF-κB dynamics in cells exposed to a pulse of TNF or lipopolysaccharide showed that cellular responses are well determined by the product of stimulus duration and concentration (Kellogg et al., 2015, King et al., 2008, Lee et al., 2016). We therefore asked whether the AUC of nuclear FP-RelA dynamics also integrates the concentration and duration of stimulus in response to a TNF ramp.

**Figure 5:**
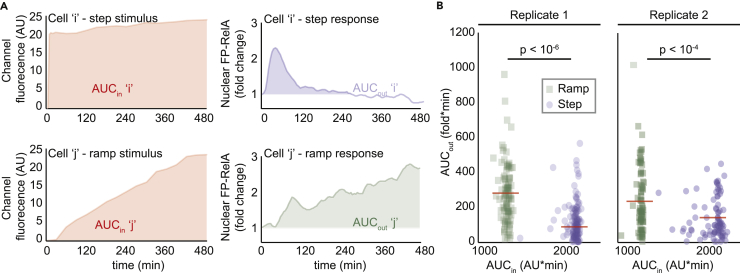
Ramp Stimulation Produces Greater Responses Despite Smaller Aggregate TNF Exposure (A) For each cell, AUC of TNF exposure (AUC_in_, left column) and fold change AUC of nuclear FP-RelA response (AUC_out_, right column) are calculated. (B) Scatterplots for independent biological replicate experiments of AUC_in_ (x axis) and AUC_out_ (y axis) for both 8-h experiments (Replicate 1: 138 cells step and 102 cells ramp; Replicate 2: 87 cells step and 80 cells ramp; see also Figure 4) show that although ramp stimulus has less AUC_in_, it produces a greater cellular response. Differences between distributions for step and ramp stimuli are statistically significant based on permutation test (see also Figure S8).

Contrary to expectations, AUC values for single-cell responses were significantly greater during stimulation with an 8-h TNF ramp versus step based on sampled permutation test (p < 10^−4^; Figure S8), a statistic that shuffles data to generate distributions for the null hypothesis. Because Alexa 647-conjugated BSA was combined with TNF in the same reservoir, its time-varying fluorescence was used to measure the dynamics of TNF concentration experienced by each cell. Scatterplots for the AUCs of Alexa 647 (total TNF input) and nuclear FP-RelA (total response) in the same cell showed that ramp stimulation generates stronger responses despite much smaller aggregate TNF exposure (Figure 5). Taken together, our data suggest that inflammatory pathway activation may be enhanced by the dynamic properties of a stimulus, such as rate of change for cytokine concentration.

### Model Predicts that Rates of Negative Feedback Determine Modes of NF-κB Activation

**Figure 6:**
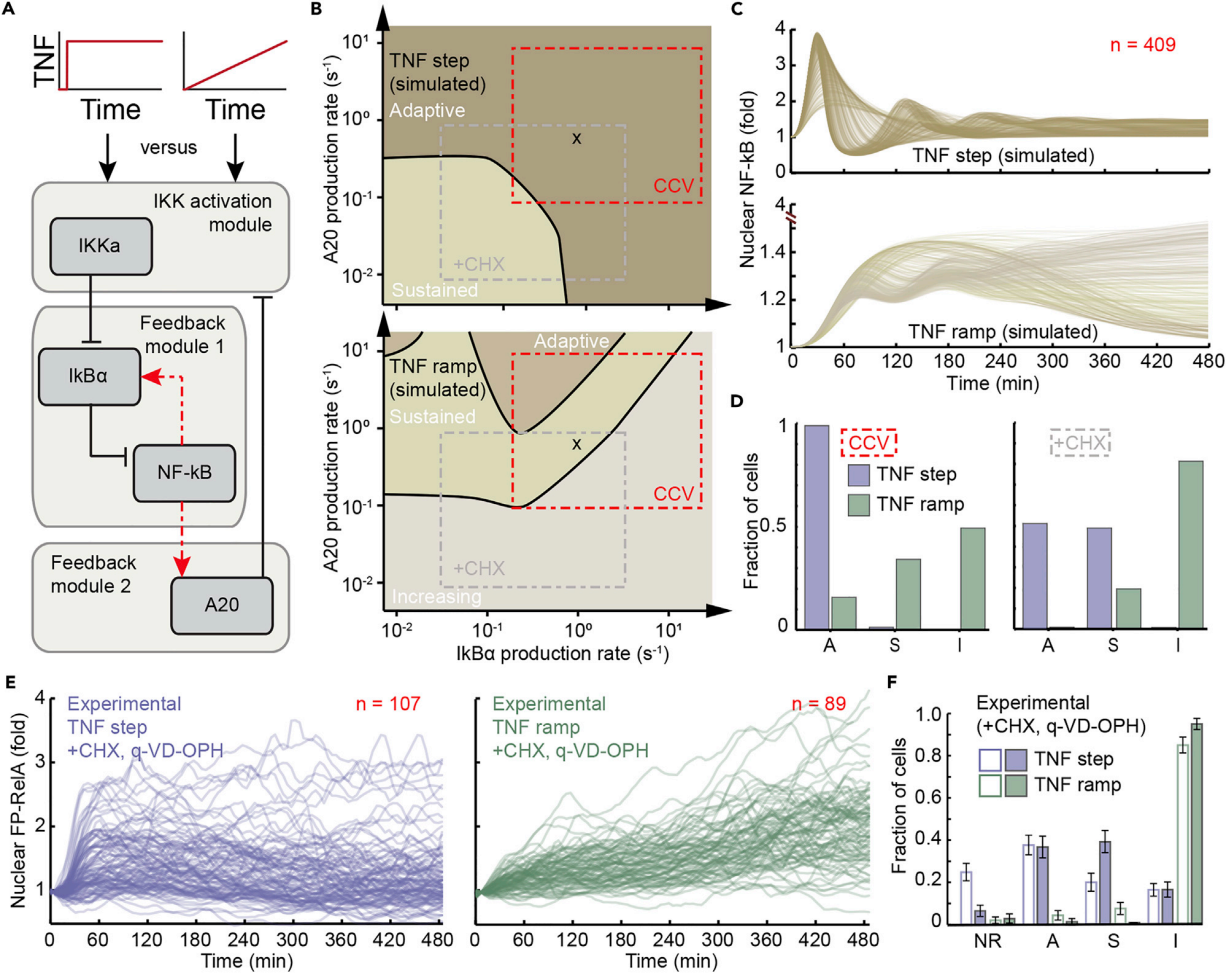
Cell-to-Cell Variability in Negative Feedback Recapitulates Response Modes (A) Schematic of negative feedback modules within the D2FC computational model (Lee et al., 2014) of the NF-κB signaling network (see also Data S3). NF-κB-driven expression of genes that encode for IkBα and A20, respectively, acts to sequester NF-κB in the cytoplasm and to limit upstream kinase activity of IKK. The activated species is denoted as IKKa in the model. (B) Simulated single-cell responses to a TNF step (top) or TNF ramp (bottom) are classified into response modes across a range of production rates for IkBα and A20 to simulate cell-to-cell variability. Although variability was modeled by simulating different translation rates for negative feedback mediators, numerically identical results can be achieved by modeling variability in transcription. The default translation rates for IkBα and A20 in the D2FC are marked with an “x.” (C) Cell-to-cell variability (CCV) is simulated by sampling values for IkBα and A20 translation rates across a range of values (red box in B; see also Transparent Methods). For each sampled pair of translation rates single-cell time course responses for a TNF step (top) or TNF ramp (bottom) are simulated. Inset number indicates number of simulated single-cell trajectories. The y axis for the simulated TNF ramp (bottom) is scaled to assist with visualization of simulated time course responses. (D) Quantification of the fraction of single cells in Adaptive (A), Sustained (S), and Increasing (I) categories for simulated single-cell trajectories in boxes marked “CCV” (left) and “+CHX” (right) in (B) (see also Transparent Methods). The +CHX box simulates cell-to-cell variability in the presence of cycloheximide to inhibit protein translation. (E) Time courses of nuclear FP-RelA fold change measured in single cells exposed to TNF stimulation as a 5-ng/mL TNF step (left) or a concentration ramp from 0 to 5 ng/mL (right) over an 8-h period for a representative experiment. CHX (640 ng/mL) and caspase inhibitor (5 μM; q-VD-OPH) are introduced to the cell culture 30 min before TNF stimulation. Inset numbers indicate the total number of cells per condition. See also Figure S9. (F) Fraction of single cells in each response class for step and ramp stimulation are enriched for sustained and increasing responses, respectively, in the presence of CHX and q-VD-OPH (c.f. Figure 4D). Independent biological replicates are shown as open and closed bars (90 and 65 cells for step and ramp conditions, respectively, in replicate 2). Distributions for step and ramp stimulation in the presence of inhibitors show statistically significant changes when compared with step and ramp distributions, respectively, in the absence of inhibitors (Figure 4D; p value < 0.00001; Pearson's chi-squared test). Error bars represent standard deviation of 5,000 bootstrap samples.

The NF-κB signaling network has been modeled as a system with two pathways of negative feedback (Figure 6A) mediated through expression of IκBα and A20 (Ashall et al., 2009, Lee et al., 2014, Tay et al., 2010). Because transcription and translation are noisy biological processes that lead to variability in protein abundances (Hansen et al., 2018), we reasoned that variability of single-cell response classes to dynamic stimuli may be a consequence of cell-to-cell variability (CCV) in negative feedback strength. Using the deterministic 2-feedback with competition (D2FC) model, which was previously parameterized to HeLa cells (Lee et al., 2014), we simulated NF-κB responses while sweeping across a broad range of parameter values for both pathways of negative feedback. For each pair of IκBα and A20 feedback parameters, single cells were simulated in response to step and ramp TNF exposure, and the resulting nuclear NF-κB time courses were classified as described for live-cell data. Although both parameter sweeps produced a non-linear response landscape, simulation results for a TNF ramp showed greater complexity and uniquely contained the “Increasing” response class in contrast with simulations for step TNF exposure (Figure 6B).

Next, to simulate the effect of variability within a population of cells, we selected a region of parameter space that allowed variation over two orders of magnitude along each axis of negative feedback, and containing the original D2FC parameterization (Figure 6B, red box “CCV”; see also Transparent Methods). Although simulated NF-κB time courses displayed long-term oscillations that are less pronounced in human cancer cell lines (Pabon et al., 2019, Wong et al., 2018, Zhang et al., 2017), and non-responsive time courses did not appear in deterministic simulations, simulated responses were otherwise qualitatively similar to live-cell data (Figures 4B and 6C). Consistent with living cells, simulated responses to step stimulation were almost exclusively “Adaptive,” whereas ramp stimulation was enriched for “Increasing” followed by “Sustained” modes of pathway activation (Figure 6D, left). However, in contrast with live-cell results, nuclear NF-κB fold change responses to a simulated ramp were smaller than step-stimulated cells (Figure 6C). AUC values comparing simulated step and ramp stimulation also show significant differences (p < 10^−6^; permutation test), but with opposite trends to experimental observations (Figure S8C), suggesting that additional differences exist between the architecture of the D2FC and mechanisms in living cells. Taken together, simulations identify rates of transcription or protein translation as determinants for different response modes in single cells exposed to dynamic stimuli.

Comparing simulations for step and ramp stimulation suggests a perturbation such as cycloheximide (CHX) that reduces rates for protein translation will respectively enrich for “Sustained” and “Increasing” response modes (boxes marked + CHX in Figures 6B and 6D, right panel; see also Transparent Methods). To test this model prediction, we exposed cells to a TNF step in a 96-well plate. Although combined TNF and CHX stimulation induces death in a fraction of cells, surviving cells showed increasingly sustained nuclear FP-RelA responses to TNF over a 5-h window in proportion with CHX concentration (Figure S9). Based on the calibration experiment, cells were exposed to a TNF step or ramp in the dynamic stimulation system in the presence of 640 ng/mL CHX in addition to quinoline-val-asp-difluorophenoxymethyl ketone (q-VD-OPH), a pan-caspase inhibitor that prevents apoptotic cell death (Figure 6E). Caspase inhibition is immunosuppressive and prevents nuclear RelA accumulation in human T cells (Lawrence and Chow, 2012), whereas caspase inhibition showed only a partial effect and nuclear FP-RelA mobilization was still observed in HeLa cells. Consistent with model predictions, the response to a TNF step switched from predominantly “Adaptive” to a nearly equal combination of “Adaptive” and “Sustained’ in the presence of inhibitors (Figure 6F; c f. Figure 4D). Similarly, after inhibition of protein translation nearly all cells exposed to a TNF ramp showed “Increasing” behavior with a reduced fraction of cells with “Adaptive” and “Sustained” responses as predicted by simulations (Figure 6F). Overall, by taking advantage of live-cell data and models, cell-to-cell variability in rates for expression of negative feedback mediators is identified as a likely contributor to behavioral diversity in cells exposed to dynamic stimuli.

## Discussion

Dynamic patterns of stimulation are necessary to probe the capabilities of signal transduction pathways and to understand how dynamic biological events modulate cellular behaviors *in vivo*. In this work, we have developed a microfluidic dynamic stimulation system for user-defined control of extracellular microenvironments. Circumventing the conventional pumping and control apparatus, our system uses gravity alone to provide precise control of stimulus dynamics. Furthermore, the system can be built from inexpensive and commonly available parts. The low operating pressures in our gravity-driven system result in minimal shear forces in the cell culture channel and are compatible with long-term cell viability during imaging experiments. Because the system's design is modular, the attached device for an experiment can be selected to provide particular capabilities such as broad dynamic range or independent control for multiple stimuli among other possibilities. Complementary to optogenetic approaches that isolate and perturb spatiotemporal dynamics of molecular signals (Tischer and Weiner, 2014, Toettcher et al., 2013), our system probes the spatiotemporal dynamics of receptor-ligand interactions or drug-response mechanisms in single cells.

Device fabrication in addition to the presence of cells and cell culture reagents can all lead to subtle variability between predicted and actual experimental results. Fluorescent dyes are useful tools to evaluate the quality of an experiment within the dynamic stimulation system. In our case, the bioactive form of TNF is trimeric with a molecular weight of 55 kDa (Smith and Baglioni, 1987), and Alexa 647-conjugated BSA was selected to mark the TNF-containing laminar stream because it has a similar molecular weight and diffusive properties. Although TNF slowly dissociates into monomers over a timescale of days when stored at low concentrations (Corti et al., 1992, Narhi and Arakawa, 1987), this is not expected to affect our results. Inside the dynamic stimulation system, TNF is stored at high concentrations and diluted within minutes of exposure to cells, effectively minimizing dissociation of TNF at low concentrations. Monomers that dissociate within this timescale will extend beyond the Alexa 647-BSA laminar boundary (Figure S2C) owing to its increased diffusivity. However, monomeric TNF denatures rapidly (Corti et al., 1992, Krippner-Heidenreich et al., 2008) and will not have biological activity in these regions of the device. Subsequent studies using similar dynamic stimulation should carefully choose tracer dyes that appropriately reproduce characteristics of ligands or molecules in the flow chamber.

When compared with step stimulation, dynamic stimulation revealed uncharacterized response classes and enhanced activation of NF-κB signaling despite smaller TNF exposure in the proof-of-concept live cell experiments. Our computational model and experiments with CHX suggest that distributions of NF-κB response modes culminate from biological noise that influences the strength of negative feedback pathways in each cell. Production and decay rates for mRNA and protein govern their abundance within a cell and are subject to varying amounts of gene-specific noise (Hansen et al., 2018, Hausser et al., 2019). Age, stress, and cell cycle phase are a few of the many contextual factors of single cells that affect reaction rates within the central dogma (Gonskikh and Polacek, 2017, Yan et al., 2016) and are likely to contribute to cell-to-cell variability of response modes to dynamic stimuli. Although our model did not show enhanced activation of NF-κB signaling in ramp-stimulated cells, we surmise that a molecular circuit upstream of inhibitor of κB kinase (IKK) activation is missing from the D2FC and other models. Indeed, studies of cytokine-dependent refractory states continue to uncover molecular determinants that are required to capture the behavior of mammalian cells that are not already reflected in models of NF-κB signaling (Adamson et al., 2016, DeFelice et al., 2019). Subsequent studies may reconcile these observations by investigating the molecular architecture and parametric constraints in the signaling network that reconstitute these missing capabilities. We expect that other well-studied signaling systems will also benefit from characterization under the lens of dynamic stimuli, using the dynamic stimulation system developed here to probe signaling networks with detailed experiments.

Cell-to-cell variability is an essential challenge in biological science laboratories and in the clinic. Probing signal transduction machinery with dynamic stimulus will reveal input-output relationships and the influence of variability within cell populations. Furthermore, our results suggest that pharmacologic control of input signal dynamics may effectively modulate the degree and mode of pathway activation for these signaling networks, which may prove valuable in designing optimal point-of-care strategies. As systems-level studies pursue subjects with increasing complexity, for example, using asynchronous perturbations to shift and exploit cell phenotypes (Lee et al., 2012, Xia et al., 2014), biology laboratories will require tools that reproducibly control dynamic cellular microenvironments. We expect that the capabilities enabled here through the low-cost, scalable, and modular dynamic stimulation system will reveal molecular mechanisms of complex cellular behaviors and can be extended generally for automation of other fluidic applications.

### Limitations of the Study

The dynamic stimulation system developed here generates precise user-defined cytokine profiles; however, the system is susceptible to common limitations associated with microfluidics. Air bubbles that form within tygon tubing and PDMS microfluidic chambers over time during flow conditions are particularly detrimental to an experiment because they cause unpredictable flow conditions. Although we routinely run successful dynamic stimulation experiments, due to this limitation long-term time courses in the order of 5 h or longer are technically challenging, which impacts experimental throughput. Another notable limitation is the design of the current proof-of-principle cell culture device that contains only a single experimental chamber. Although the gravity pump can theoretically multiplex control over several experiments at the same time, only one dynamic stimulation condition can be observed in a single experiment with the proof-of-principle device. In future iterations, design alterations that restrict air bubbles and incorporate multiple cell culture chambers will increase consistency of long-term time courses and increase throughput by simultaneously controlling experiments in parallel.

## Methods

All methods can be found in the accompanying Transparent Methods supplemental file.

## Supplemental Information

Document S1. Transparent Methods and Figures S1–S9

Data S1. Stereolithography File to 3D-Print Mold for the Dynamic Stimulation Device, Related to Figure 1 Stereolithography (.stl) file used to 3D-print molds for the dynamic stimulation device.

Data S2. Stereolithography Files to 3D-Print Inlet and Outlet Reservoirs, Related to Figure 1 Stereolithography (.stl) file used to 3D-print inlet and outlet reservoir basins used with the gravity pump.

Data S3. D2FC Computational Model, Related to Figure 6 MATLAB files for the D2FC model of the NF-κB are provided.

## References

[bib1] Adamson A., Boddington C., Downton P., Rowe W., Bagnall J., Lam C., Maya-Mendoza A., Schmidt L., Harper C.V., Spiller D.G. (2016). Signal transduction controls heterogeneous NF-kappaB dynamics and target gene expression through cytokine-specific refractory states. Nat. Commun..

[bib2] Albeck J.G., Mills G.B., Brugge J.S. (2013). Frequency-modulated pulses of ERK activity transmit quantitative proliferation signals. Mol. Cell.

[bib3] Ashall L., Horton C.A., Nelson D.E., Paszek P., Harper C.V., Sillitoe K., Ryan S., Spiller D.G., Unitt J.F., Broomhead D.S. (2009). Pulsatile stimulation determines timing and specificity of NF-kB – dependent transcription. Science.

[bib4] Bennett M.R., Pang W.L., Ostroff N.A., Baumgartner B.L., Nayak S., Tsimring L.S., Hasty J. (2008). Metabolic gene regulation in a dynamically changing environment. Nature.

[bib5] Brimmo A., Goyette P.A., Alnemari R., Gervais T., Qasaimeh M.A. (2018). 3D printed microfluidic probes. Sci. Rep..

[bib6] Corti A., Fassina G., Marcucci F., Barbanti E., Cassani G. (1992). Oligomeric tumour necrosis factor alpha slowly converts into inactive forms at bioactive levels. Biochem. J..

[bib7] DeFelice M.M., Clark H.R., Hughey J.J., Maayan I., Kudo T., Gutschow M.V., Covert M.W., Regot S. (2019). NF-kappaB signaling dynamics is controlled by a dose-sensing autoregulatory loop. Sci. Signal..

[bib8] Fullerton J.N., Gilroy D.W. (2016). Resolution of inflammation: a new therapeutic frontier. Nat. Rev. Drug Discov..

[bib9] Gomez-Sjoberg R., Leyrat A.A., Pirone D.M., Chen C.S., Quake S.R. (2007). Versatile, fully automated, microfluidic cell culture system. Anal. Chem..

[bib10] Gonskikh Y., Polacek N. (2017). Alterations of the translation apparatus during aging and stress response. Mech. Ageing Dev..

[bib11] Hansen M.M.K., Desai R.V., Simpson M.L., Weinberger L.S. (2018). Cytoplasmic amplification of transcriptional noise generates substantial cell-to-cell variability. Cell Syst..

[bib12] Hausser J., Mayo A., Keren L., Alon U. (2019). Central dogma rates and the trade-off between precision and economy in gene expression. Nat. Commun..

[bib13] He L., Kniss A., San-Miguel A., Rouse T., Kemp M.L., Lu H. (2015). An automated programmable platform enabling multiplex dynamic stimuli delivery and cellular response monitoring for high-throughput suspension single-cell signaling studies. Lab Chip.

[bib14] Hersen P., McClean M.N., Mahadevan L., Ramanathan S. (2008). Signal processing by the HOG MAP kinase pathway. Proc. Natl. Acad. Sci. U S A.

[bib15] Ho C.M., Ng S.H., Li K.H., Yoon Y.J. (2015). 3D printed microfluidics for biological applications. Lab Chip.

[bib16] Hoffmann A., Levchenko A., Scott M.L., Baltimore D. (2002). The IkappaB-NF-kappaB signaling module: temporal control and selective gene activation. Science.

[bib17] Hsieh H.J., Liu C.A., Huang B., Tseng A.H., Wang D.L. (2014). Shear-induced endothelial mechanotransduction: the interplay between reactive oxygen species (ROS) and nitric oxide (NO) and the pathophysiological implications. J. Biomed. Sci..

[bib18] Kellogg R.A., Tian C., Lipniacki T., Quake S.R., Tay S. (2015). Digital signaling decouples activation probability and population heterogeneity. Elife.

[bib19] Kim S.J., Yokokawa R., Lesher-Perez S.C., Takayama S. (2012). Constant flow-driven microfluidic oscillator for different duty cycles. Anal. Chem..

[bib20] King K.R., Wang S., Jayaraman A., Yarmush M.L., Toner M. (2008). Microfluidic flow-encoded switching for parallel control of dynamic cellular microenvironments. Lab Chip.

[bib21] Krippner-Heidenreich A., Grunwald I., Zimmermann G., Kuhnle M., Gerspach J., Sterns T., Shnyder S.D., Gill J.H., Mannel D.N., Pfizenmaier K. (2008). Single-chain TNF, a TNF derivative with enhanced stability and antitumoral activity. J. Immunol..

[bib22] Kuczenski B., LeDuc P.R., Messner W.C. (2007). Pressure-driven spatiotemporal control of the laminar flow interface in a microfluidic network. Lab Chip.

[bib23] Kumar R., Clermont G., Vodovotz Y., Chow C.C. (2004). The dynamics of acute inflammation. J. Theor. Biol..

[bib24] Lawrence C.P., Chow S.C. (2012). Suppression of human T cell proliferation by the caspase inhibitors, z-VAD-FMK and z-IETD-FMK is independent of their caspase inhibition properties. Toxicol. Appl. Pharmacol..

[bib25] Lee P.J., Helman N.C., Lim W.A., Hung P.J. (2008). A microfluidic system for dynamic yeast cell imaging. Biotechniques.

[bib26] Lee P.J., Gaige T.A., Hung P.J. (2009). Dynamic cell culture: a microfluidic function generator for live cell microscopy. Lab Chip.

[bib27] Lee M.J., Ye A.S., Gardino A.K., Heijink A.M., Sorger P.K., MacBeath G., Yaffe M.B. (2012). Sequential application of anticancer drugs enhances cell death by rewiring apoptotic signaling networks. Cell.

[bib28] Lee R.E., Walker S.R., Savery K., Frank D.A., Gaudet S. (2014). Fold change of nuclear NF-kappaB determines TNF-induced transcription in single cells. Mol. Cell.

[bib29] Lee R.E., Qasaimeh M.A., Xia X., Juncker D., Gaudet S. (2016). NF-kappaB signalling and cell fate decisions in response to a short pulse of tumour necrosis factor. Sci. Rep..

[bib31] Mavrogiannis N., Ibo M., Fu X., Crivellari F., Gagnon Z. (2016). Microfluidics made easy: a robust low-cost constant pressure flow controller for engineers and cell biologists. Biomicrofluidics.

[bib32] Medzhitov R. (2008). Origin and physiological roles of inflammation. Nature.

[bib33] Mehling M., Tay S. (2014). Microfluidic cell culture. Curr. Opin. Biotechnol..

[bib34] Nagel T., Resnick N., Dewey C.F., Gimbrone M.A. (1999). Vascular endothelial cells respond to spatial gradients in fluid shear stress by enhanced activation of transcription factors. Arterioscler. Thromb. Vasc. Biol..

[bib35] Narhi L.O., Arakawa T. (1987). Dissociation of recombinant tumor necrosis factor-alpha studied by gel permeation chromatography. Biochem. Biophys. Res. Commun..

[bib36] Pabon N.A., Zhang Q., Cruz J.A., Schipper D.L., Camacho C.J., Lee R.E.C. (2019). A network-centric approach to drugging TNF-induced NF-kappaB signaling. Nat. Commun..

[bib37] Piehler A., Ghorashian N., Zhang C., Tay S. (2017). Universal signal generator for dynamic cell stimulation. Lab Chip.

[bib38] Purvis J.E., Karhohs K.W., Mock C., Batchelor E., Loewer A., Lahav G. (2012). p53 dynamics control cell fate. Science.

[bib39] Purvis J.E., Lahav G. (2013). Encoding and decoding cellular information through signaling dynamics. Cell.

[bib40] Ryu H., Chung M., Dobrzynski M., Fey D., Blum Y., Sik Lee S., Peter M., Kholodenko B.N., Li Jeon N., Pertz O. (2016). Frequency modulation of ERK activation dynamics rewires cell fate. Mol. Syst. Biol..

[bib41] Ryu H., Chung M., Song J., Lee S.S., Pertz O., Jeon N.L. (2018). Integrated platform for monitoring single-cell MAPK kinetics in computer-controlled temporal stimulations. Sci. Rep..

[bib42] Smith R.A., Baglioni C. (1987). The active form of tumor necrosis factor is a trimer. J. Biol. Chem..

[bib43] Spencer S.L., Gaudet S., Albeck J.G., Burke J.M., Sorger P.K. (2009). Non-genetic origins of cell-to-cell variability in TRAIL-induced apoptosis. Nature.

[bib44] Takayama S., Ostuni E., LeDuc P., Naruse K., Ingber D.E., Whitesides G.M. (2003). Selective chemical treatment of cellular microdomains using multiple laminar streams. Chem. Biol..

[bib45] Tay S., Hughey J.J., Lee T.K., Lipniacki T., Quake S.R., Covert M.W. (2010). Single-cell NF-kappaB dynamics reveal digital activation and analogue information processing. Nature.

[bib46] Tischer D., Weiner O.D. (2014). Illuminating cell signalling with optogenetic tools. Nat. Rev. Mol. Cell Biol..

[bib47] Toettcher J.E., Weiner O.D., Lim W.A. (2013). Using optogenetics to interrogate the dynamic control of signal transmission by the Ras/Erk module. Cell.

[bib48] Whitesides G.M. (2006). The origins and the future of microfluidics. Nature.

[bib49] Wong V.C., Bass V.L., Bullock M.E., Chavali A.K., Lee R.E.C., Mothes W., Gaudet S., Miller-Jensen K. (2018). NF-kappaB-chromatin interactions drive diverse phenotypes by modulating transcriptional noise. Cell Rep..

[bib50] Xia X., Owen M.S., Lee R.E.C., Gaudet S. (2014). Cell-to-cell variability in cell death: can systems biology help us make sense of it all?. Cell Death Dis..

[bib51] Yan X., Hoek T.A., Vale R.D., Tanenbaum M.E. (2016). Dynamics of translation of single mRNA molecules in vivo. Cell.

[bib52] Zambrano S., De Toma I., Piffer A., Bianchi M.E., Agresti A. (2016). NF-kappaB oscillations translate into functionally related patterns of gene expression. Elife.

[bib53] Zhang Q., Gupta S., Schipper D.L., Kowalczyk G.J., Mancini A.E., Faeder J.R., Lee R.E.C. (2017). NF-kappaB dynamics discriminate between TNF doses in single cells. Cell Syst..

