# Supplementary material for: A System for Analog Control of Cell Culture Dynamics to Reveal Capabilities of Signaling Networks
Source: iScience. 2019 Aug 8;19:586–96. doi: 10.1016/j.isci.2019.08.010 (PMC6713801; doi:10.1016/j.isci.2019.08.010)
Supplement: Document S1. Transparent Methods and Figures S1–S9 [file mmc1.pdf]

ISCI, Volume *19*

Supplemental Information

A System for Analog Control of Cell Culture Dynamics to Reveal Capabilities of Signaling Networks

Chaitanya S. Mokashi, David L. Schipper, Mohammad A. Qasaimeh, and Robin E.C. Lee

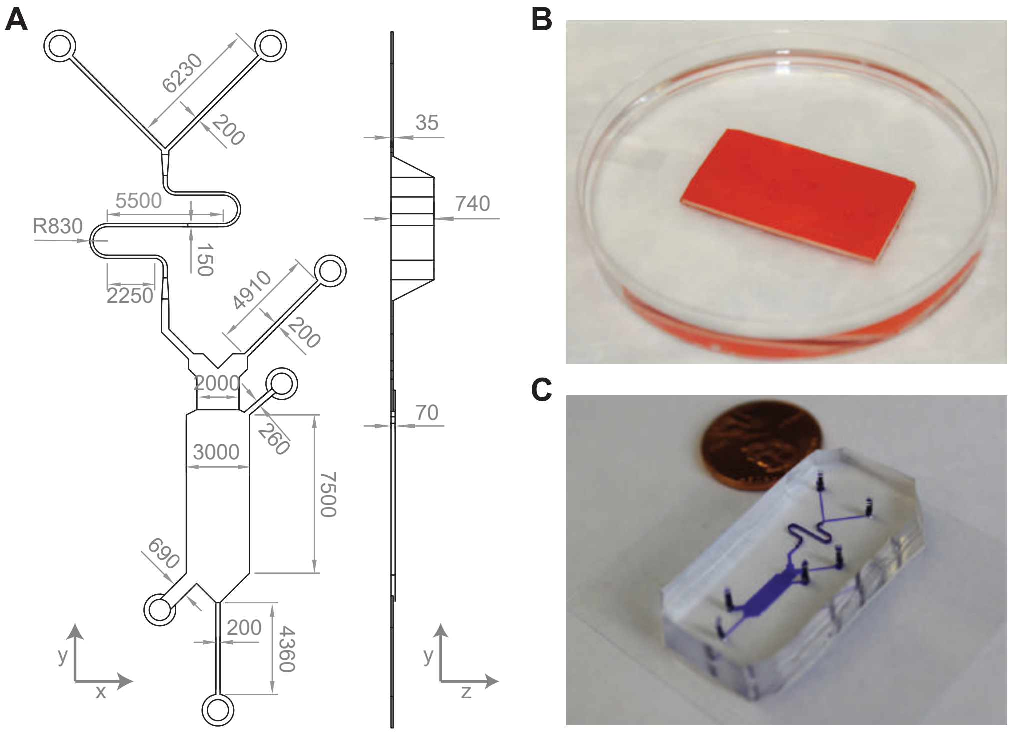

Figure S1. Design and fabrication of dynamic stimulation device, Related to Figure 1. **(A)** Top-down (left) and relief (right) views of the dynamic stimulation device. All dimensions are in micrometers. **(B)** The 3D-printed mold is pasted to a petri dish and covered with PDMS mixed with a hardening agent. The cured PDMS relief is cut along the edges of the mold with a box cutter, carefully peeled from the mold, and bonded with a glass cover slip. **(C)** The completed device filled with a dye for visualization, coin provided for scale.

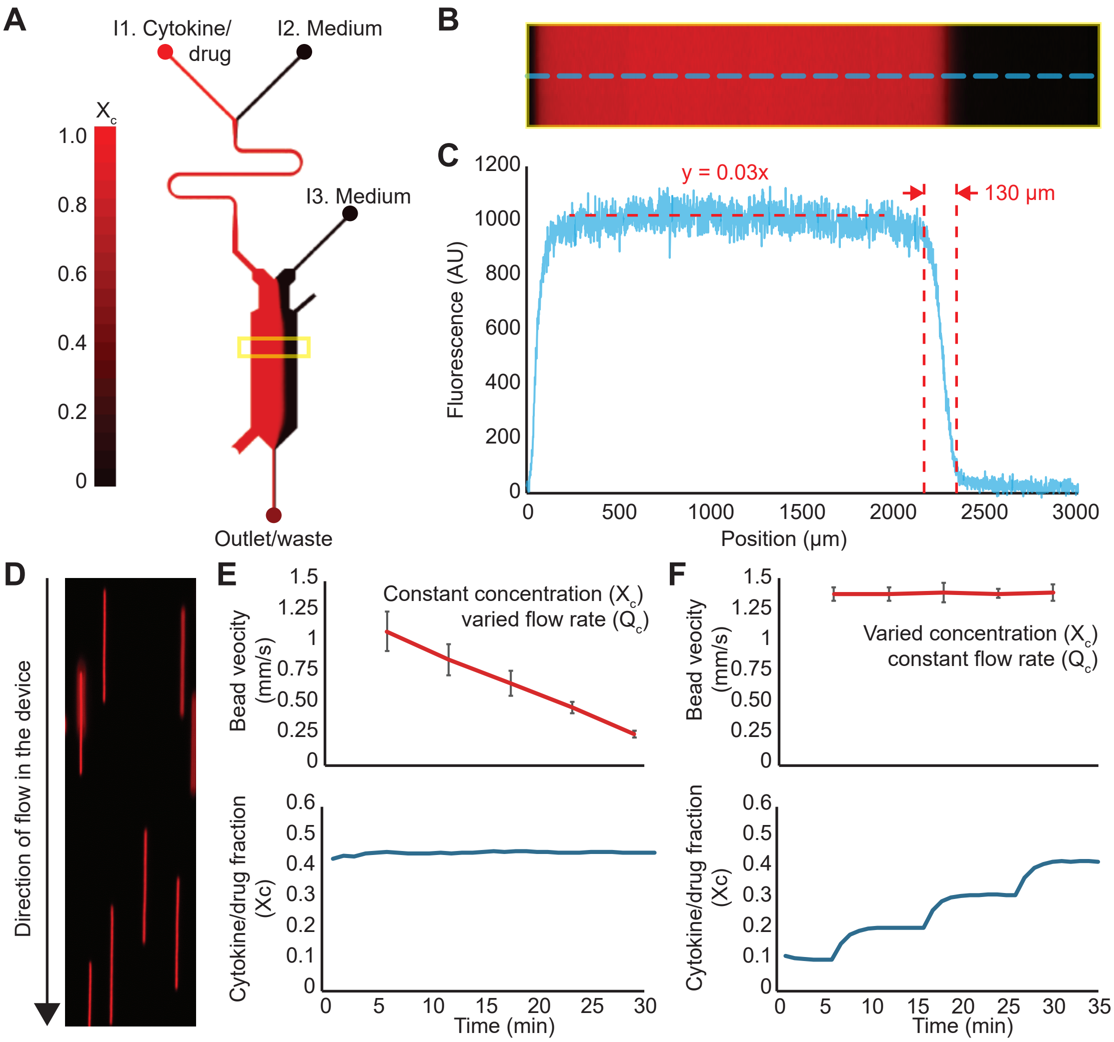

Figure S2. Characterization of mixing and flow rates within in the dynamic stimulation device, Related to Figure 2. (A) Representative Ansys-Fluent simulation of the device with volume fraction of stimulus at inlet I1 is set to 1 and that at inlets I2 and I3 is set to 0. Many such simulations were performed with different inlet pressures (P1, P2 and P3) and corresponding inlet flow rates (Q1, Q2 and Q3) were used to fit the linear model. **(B)** Alexa647-conjugated BSA intensity was diluted through the mixer and a panel of images were collected across a cross-section of the cell culture channel (depicted by yellow box in panel a). **(C)** Representative fluorescence of Alexa647-conjugated BSA was collected along a line scan (blue dotted line in panel B) shows an even distribution across 'M' and 'E' bands of the channel demonstrating nearly perfect mixing with a small diffusive region (< 150 µm) between the 'E' and 'C' bands. **(D)** Path lines of fluorescent tracer beads (FluoSpheres) used to measure the flow rate in the cell culture channel. Bead velocity is calculated by dividing the trace length by the exposure time of the microscope. **(E)** The flow rate, measured by bead velocity, can be independently controlled while keeping the concentration (Xc) of Alexa647-conjugated BSA unchanged. **(F)** Concentration of the stimulus in terms of Xc can be independently controlled while keeping the flow rate unchanged. Error bars describe the standard deviation of 40 beads per data point on average.

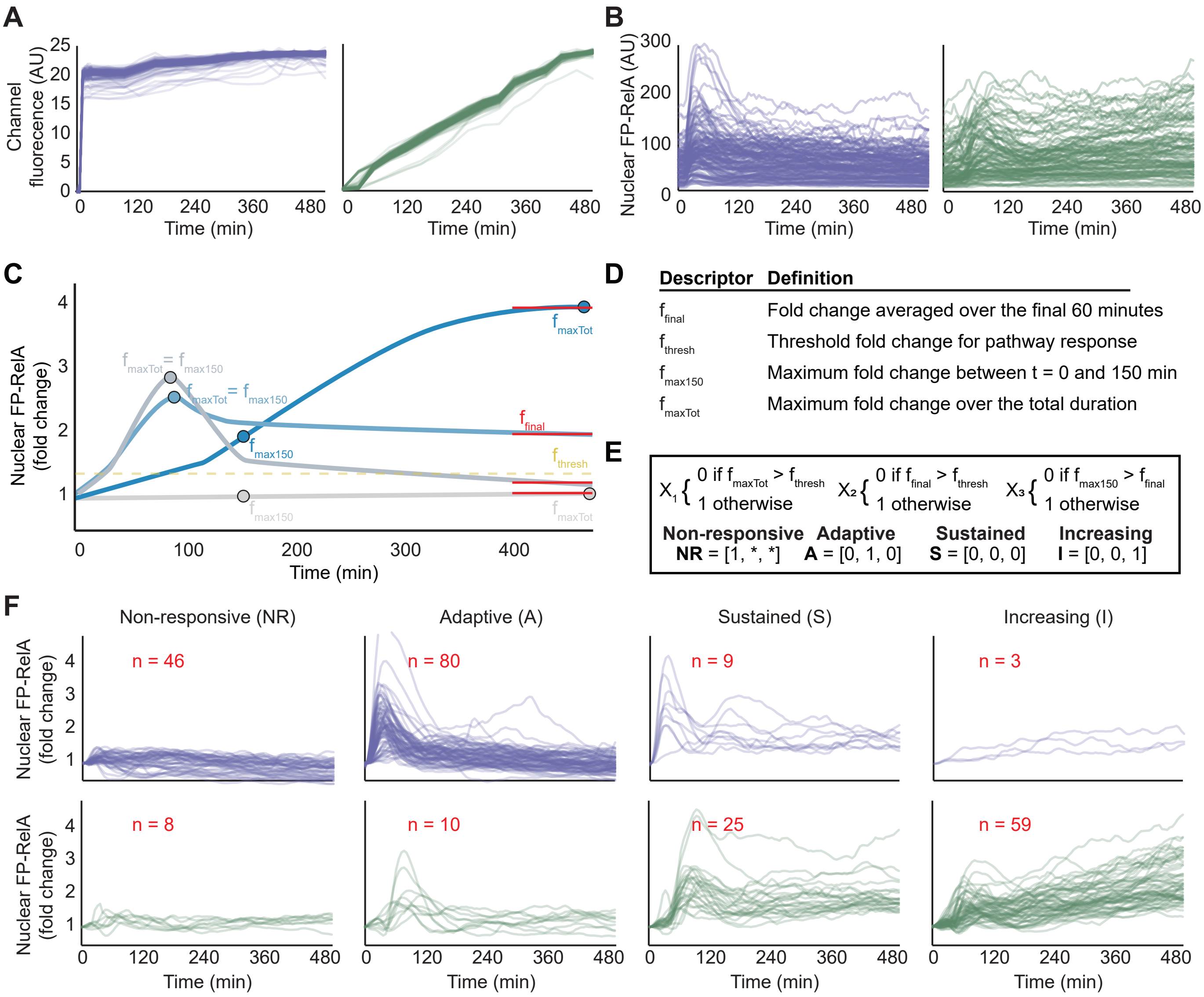

Figure S3. Categories of single-cell responses to 8h step and ramp stimulation, related to Figure 4. (A) Fluorescence intensity of Alexa 647-conjugated BSA was measured at every cell over 8 hours for step (left) and ramp (right) TNF treatment for a representative experiment. The fluorescence intensity was normalized to maximum to get fractional intensity, and used as proxy for volume fraction (Xc) of TNF concentration between 0 and 5ng/mL. **(B)** Raw time-course trajectories for nuclear FP-RelA for single cells exposed to step (left) and ramp (right) TNF conditions for a representative experiment. The same trajectories are plotted as nuclear fold change in Figure 4. **(C)** Example of quantitative descriptors extracted from illustrative single cell time courses of nuclear FP-RelA fold change, defined in **(D)**. **(E)** Each time course is represented as a vector of the form [X_1, X_2, X_3] and used to classify cells exposed to TNF as either non-responsive (NR), adaptive (A), sustained (S), and increasing (I). **(F)** Time courses of nuclear FP-RelA in (B) are classified into four categories as defined by the feature-based classification algorithm in (E). Insets show number of cells in each category for each condition.

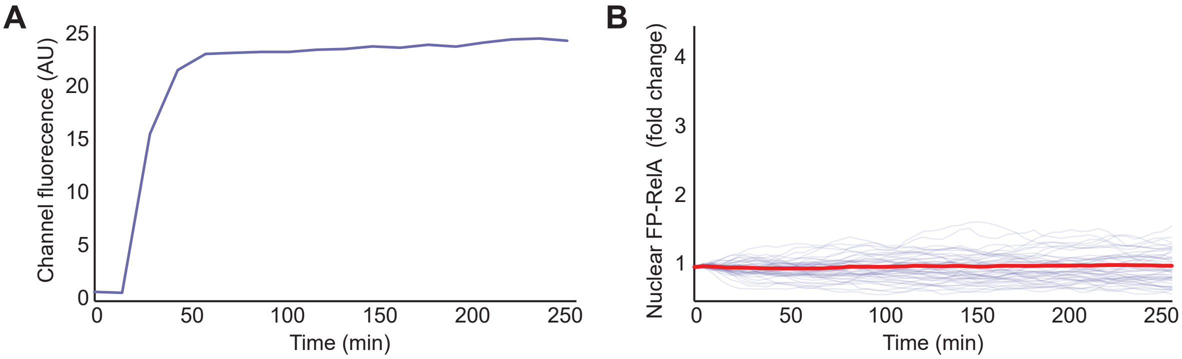

Figure S4. Responses to a mock step of Alexa 647-conjugated BSA but without TNF, Related to Figure 4. (A) Fluorescence intensity of Alexa 647-conjugated BSA was measured in the cell culture channel in an experiment during a mock treatment, where cells were exposed to a step of BSA but were not exposed to TNF. (B) Average (red line) of 46 nuclear FP-RelA single cell time courses (purple lines) do not show evidence of mechanotransduction-dependent pathway activation.

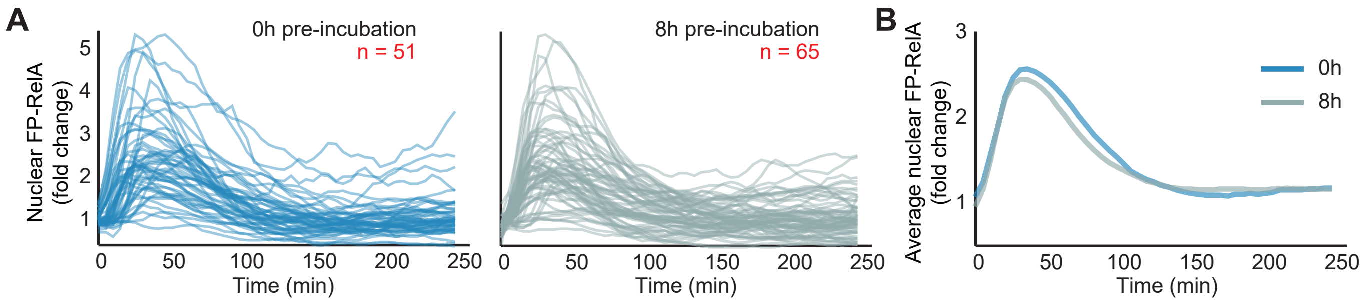

Figure S5. TNF retains bioactivity over the duration of experiments, Related to Figure 4. (A) Time-course trajectories for nuclear FP-RelA in single cells exposed to a 5 ng/mL TNF step in a 96-well plate. TNF was prepared either immediately before cell stimulation (left, 0h) or 8-hours before cell stimulation (right, 8h) and incubated at room temperature. Red inset numbers indicate the number of single cell trajectories collected in each condition. **(B)** Average of single cell time-courses for nuclear FP-RelA demonstrate that TNF retains comparable bioactivity over an 8-hour duration.

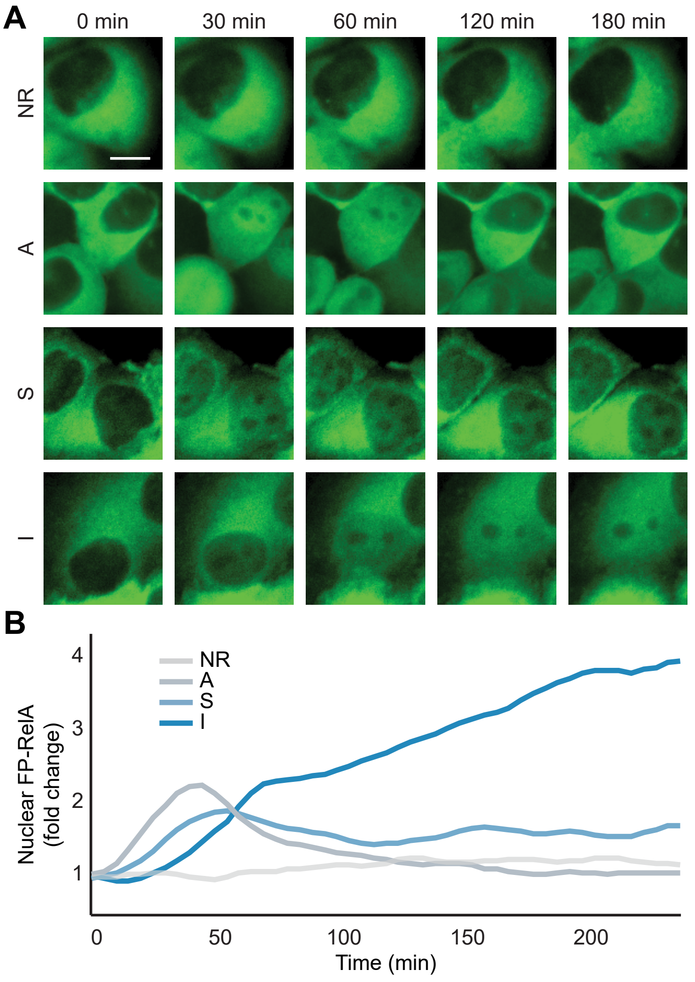

Figure S6. Example single cell trajectories for nuclear FP-RelA response categories, Related to Figure 4. (A) Time-lapse images of FP-RelA-expressing HeLa cells illustrate non-responsive (NR), adaptive (A), sustained (S), and increasing (I) classifications in cells exposed to TNF. Scale bar is 10 μm. **(B)** Quantification of nuclear FP-RelA from single cells in panel A).

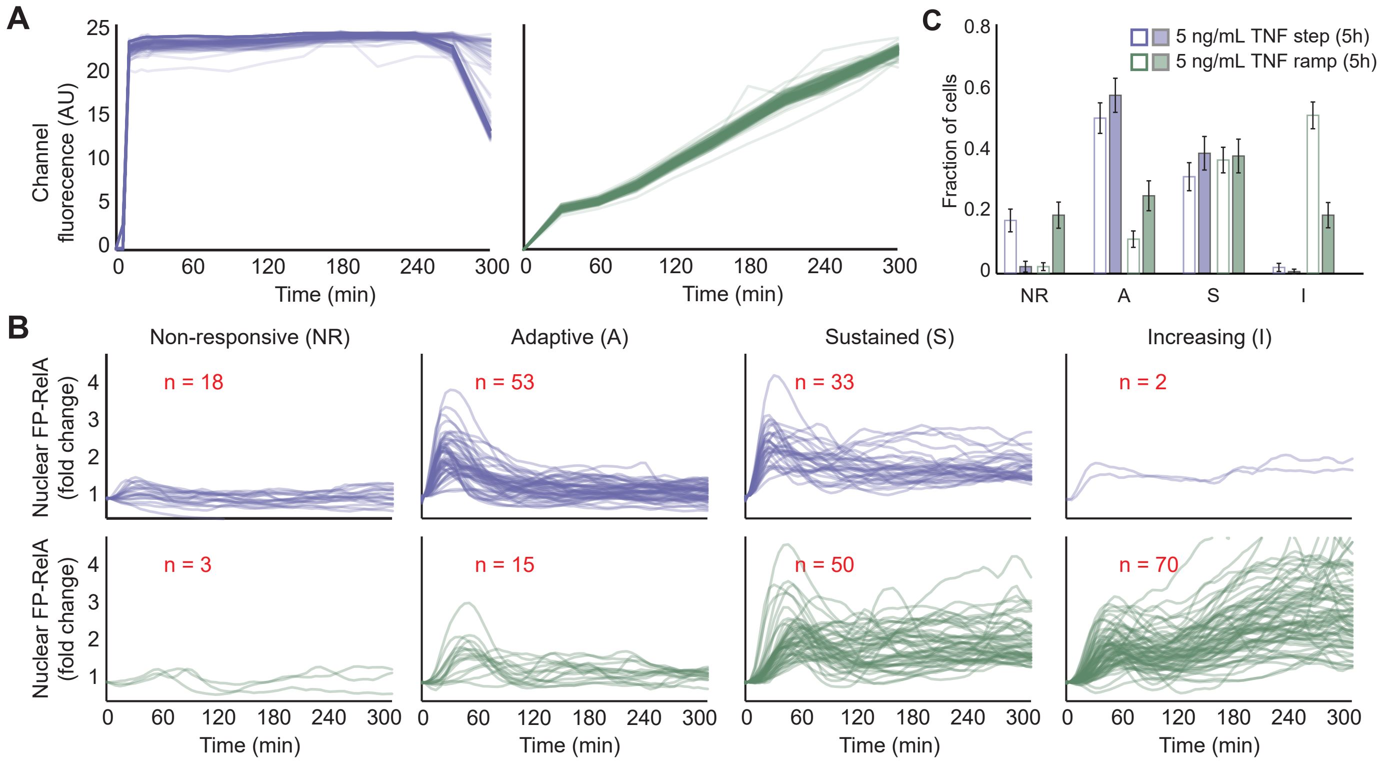

Figure S7. Responses to a 5-hour exposure to step or ramped TNF stimulation, related to Figure 4. Fluorescence intensity of Alexa 647-conjugated BSA was measured at every cell over 5 hours for **(A)** step (left) and ramp (right) TNF treatment in a representative experiment. The fluorescence intensity was normalized to maximum to get fractional intensity, and used as proxy for volume fraction (Xc) of TNF concentration between 0 and a maximum of 5ng/mL. **(B)** Time courses of nuclear FP-RelA are classified into four categories: Non-responsive, Adaptive, Sustained, and Increasing, for continuous (top row) and ramp (bottom row) stimulation with TNF for a representative experiment. Insets show number of cells in each category for both conditions. **(C)** Quantification of the fraction of single cells in each category for 5-hour step and ramp stimulation shows show statistically significant differences in their distributions (p-value = 0.05; Pearson's chi-squared test). Biological duplicate data are shown as open and closed barss and show enrichment of the Increasing category after ramp stimulation as observed for an 8-hour ramp in Figure 4. Error bars represent standard deviation of 5000 bootstrap samples.

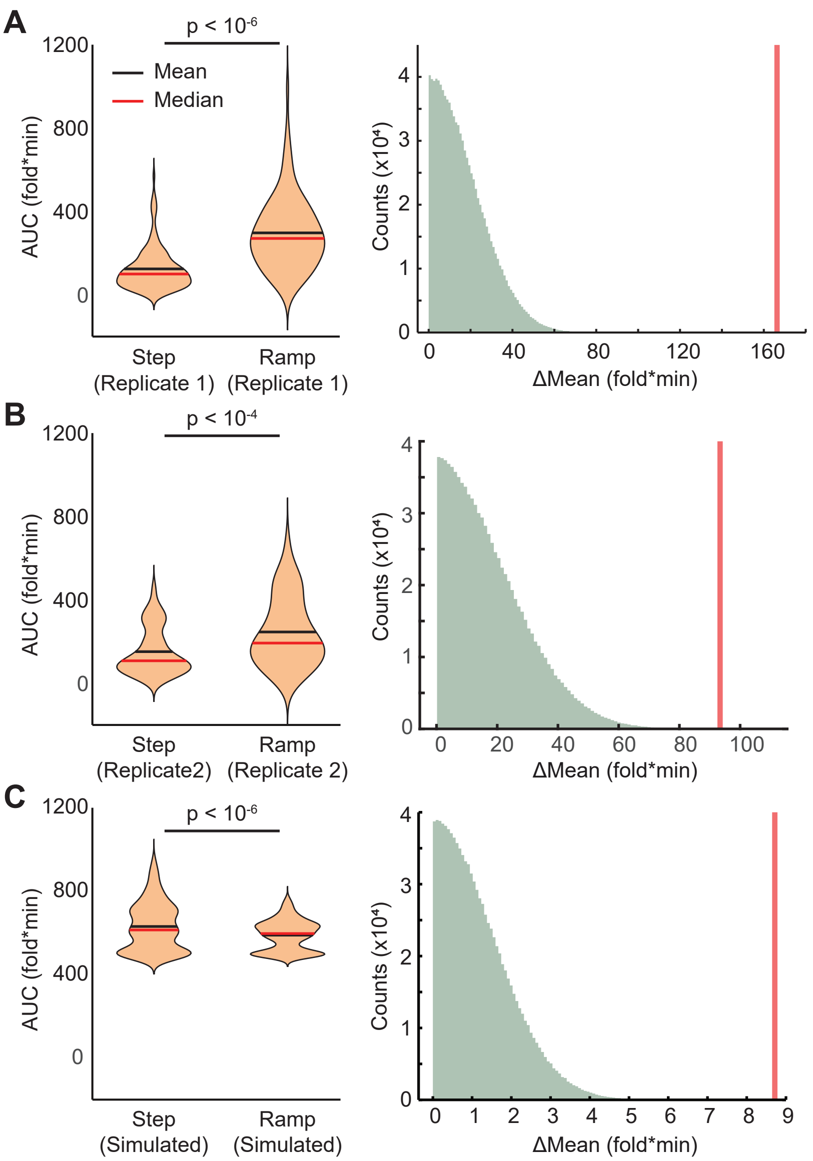

Figure S8. Ramp stimulation produces greater AUC responses, related to Figure 5. For each cell, the area under the curve (AUC) of nuclear FP-RelA response is calculated. Violin plots (left) and permutation test (right) showing a significant difference between the AUCs of fold change responses between 8h step and ramp stimuli for **(A)** biological replicate 1, **(B)** biological replicate 2, and **(C)** simulated data (see figure 6). For permutation tests, AUC data from a step and ramp stimulation experiment are randomly permuted between groups without replacement and the difference between the means (ΔMean) of the permuted data is measured. Frequency histograms (right) depict ΔMean of the AUC for 10^6 permutations of the data. Red lines indicate the ΔMean of the original unpermuted data set. For (A) and (C), values of ΔMean for all permuted data sets are smaller than the ΔMean of the original unpermuted data demonstrating statistical significance with a p-value $< 10^{-6}$ (two-tailed). For (B) the two-tailed p-value $< 10^{-4}$. We therefore reject the null hypothesis and accept the alternative for all three tests, that differences between the AUC of step and ramp data did not occur by chance.

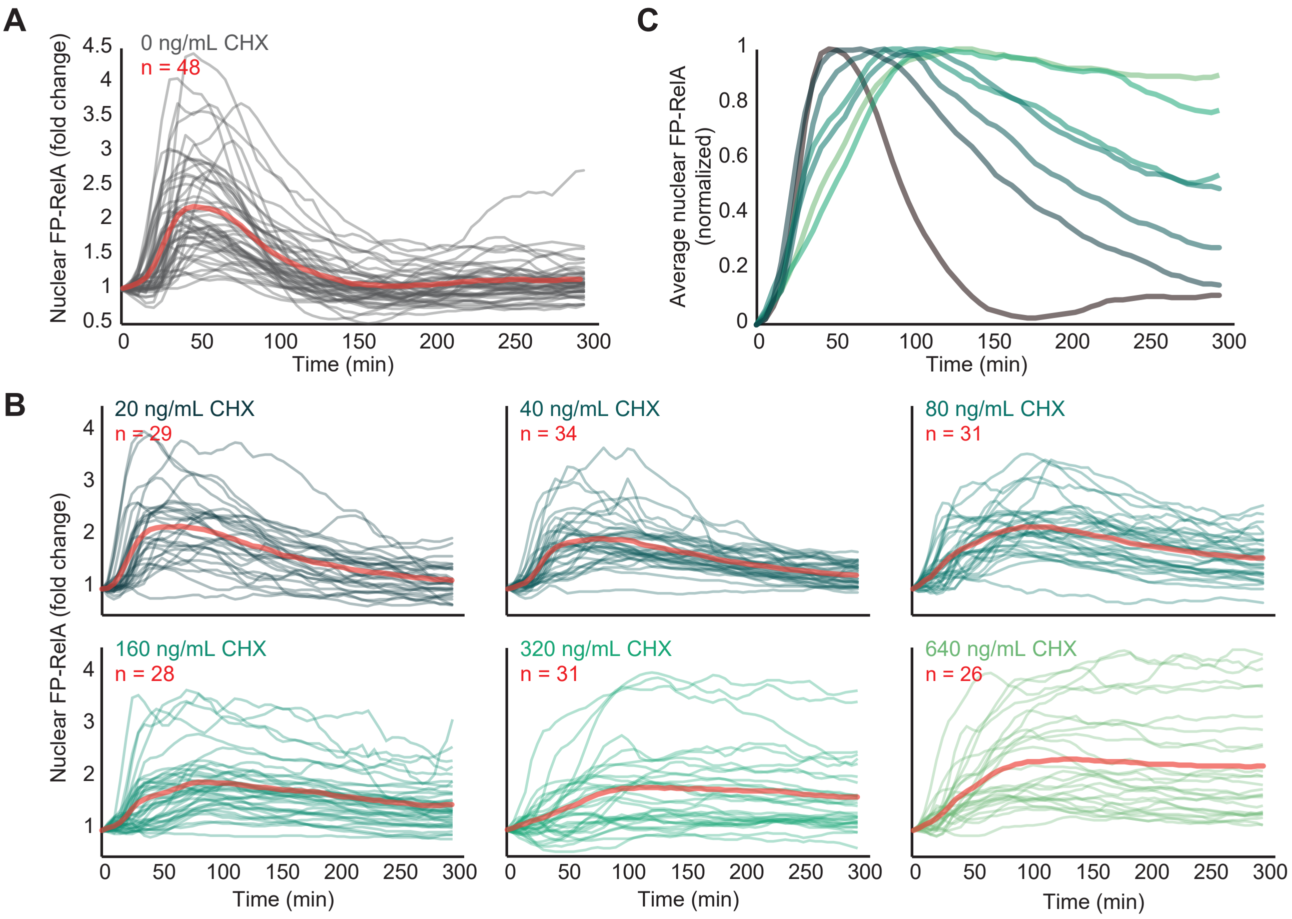

Figure S9. Nuclear FP-RelA trajectories in response to a TNF step are sustained in the presence of cycloheximide, related to Figure 6. (A) Time-course trajectories for nuclear FP-RelA in single cells exposed to a 5 ng/mL TNF step. Red line indicates the average of single cell responses. **(B)** Time-course trajectories for nuclear FP-RelA in single cells exposed to a 5 ng/mL TNF step in the presence of indicated concentration of cycloheximide (CHX, added 60 minutes before TNF stimulation). Significant cell death was observed in many conditions. Consequently trajectories were only collected from cells that survived for at least 300 minutes after TNF exposure. Red line indicates the average of single cell responses for each condition. **(C)** Average of of single cell time-courses for each condition in panels A) and B). Nuclear FP-RelA is normalized for each condition to demonstrate differences in nuclear export of FP-RelA attributed to CHX-dependent inhibition of protein translation for mediators of negative feedback. All CHX calibration experiments were performed in 96-well plates. Red inset numbers indicate the number of single cell trajectories collected in each condition.

TRANSPARENT METHODS:

Gravity pump overview

The gravity pump is controlled by a programmable Arduino mega 2560 microcontroller (Arduino). The stepper motors (generic DC 4-9V Drive Stepper Motor Screw with Nut Slider 2-Phase 4-Wire), Arduino controller, and stepper drivers (A3967 Easydriver V44) are mounted on a custom-made acrylic frame with machined holes to ensure level mounting. Each stepper motor is controlled by its own stepper driver board powered by a 9V 5A power supply. The stepper driver logic pins are connected to and controlled by the Arduino digital I/O pins. Each stepper motor assembly has a screw-nut drive platform that moves up or down vertically as per the direction of rotation of the motor. We designed and 3D-printed basins (STL designs provided in Supplemental Files) with a base allowing them to attach stably to the stepper platforms and an internal volume of 1.64ml. The basins contained ports with sufficient tolerances to allow friction fit with tygon tubes (Fisher Scientific 1471139).

Design and microfabrication of the dynamic stimulation device

Autodesk Fusion 360 CAD software (Autodesk) was used to design the molds and produce the corresponding STL design files for 3D printing (see Supplemental files). Molds were printed with Solus Proto resin using a SOLUS DLP 3D printer (Junction3D) in the high-resolution (25 µm X/Y) setting and 25 µm Z axis step size. Freshly printed molds were washed with ethanol and exposed face up to UV light for 30 minutes while submerged in ddH2O. Cured molds were adhered to a 10 cm cell culture dish with double sided tape.

PDMS (Sylgard) was prepared at a ratio of 10:1 PDMS to hardening agent and poured over the mold. Vacuum was applied to remove bubbles and the device was placed in incubator at 70 ^{0}C to cure for approximately 2 hours. Alternatively, lower temperature can be used which results in longer curing time. The hardened PDMS around the base of the mold was cut with a

box cutter and the device carefully peeled from the mold. The edges of the device were trimmed with razor blade and 1.25mm holes were punched into the inlets, outlet, and loading channels. Devices were then soaked in 70% EtOH for 30 minutes and let dry for an additional 30 minutes. The base of each device was cleaned using scotch tape to remove any dust and debris. The device was plasma bonded (Harrick Plasma, PDC001) to 45mmx50mm glass cover slip at 800mtorr pressure on the high setting for 1 min. Due to the low pressures during operation, we expect the dynamic stimulation device should be compatible with other non-plasma approaches that reversibly bond PDMS and glass (Harris et al., 2007). Cell cultures exposed to solidified resin from the 3D printer (UV-treated) continued to survive and grow with the same rates and morphology as untreated cells, suggesting that any trace residues from the fabrication process are compatible with cellular assays.

Device preparation and cell seeding

Dynamic stimulation devices made in PDMS were autoclaved and treated with UV light for disinfection. Devices were washed with ethanol and then with a solution of 0.002 v/v fibronectin in phosphate buffer saline (PBS). The devices were then incubated with the fibronectin solution for 24 hours by using 200µl pipette tips inserted in the inlets.

For cell seeding, devices coated with the fibronectin solution for 24 hours were flushed with media multiple times. All the inlets except the cell seeding ports were plugged with PDMS plugs made by hardening PDMS in 200 µl pipette tips, and a 200 µl pipette tip filled with media was inserted in the wider seeding port. Cells suspended in media between $3*10^6$ and $8*10^6$ cells/ml density were seeded through the narrower seeding port while observing under a microscope. After reaching a cell density for approximately 60% confluence, the narrower cell-seeding port was plugged with a PDMS plug and the device is incubated for at least 24 hours, allowing cells to attach and spread out on the glass surface. The PDMS plugs at the inlets and

outlet were then replaced with a 200 µl pipette tip filled with medium and further incubated until the time of experiment with daily medium changes.

Control model for the dynamic stimulation system

The three controlled variables in the model are the volume fraction of drug (Xc), the laminar interface position of the treatment stream (LP) and the total volumetric flow rate (Qc):

$$Xc = \frac{Q1}{Q1 + Q2}$$

$$LP = \frac{Q1 + Q2}{Q1 + Q2 + Q3}$$

Qc = set by the user

Where Q1, Q2 and Q3 are the inlet flow rates corresponding to the inlets I1, I2 and I3 (Figure 1A).

A linear model between the hydrostatic pressures (P1, P2 and P3) and the inlet flow rates (Q1, Q2 and Q3) is used to determine reservoir heights corresponding to the desired values of controlled variables (Xc, Lp and Qc):

$$Q = R * P$$

$$h(mm) = \frac{P}{9.8 * 1000}$$

Where **Q** is the 3x1 vector of inlet flow rates, **P** is the 3x1 vector of driving pressures, **h** is the 3x1 height vector corresponding to the hydrostatic pressures, and **R** is the 3x3 fluidic resistance matrix. Multiple CFD simulations corresponding to different combinations of the three driving pressures were carried out in ANSYS-Fluent to calculate the corresponding inlet flow rates. The linear model was then fitted to these data to estimate the resistance matrix R. A calibration experiment was run with the model output and compared with the desired input. The model was

then adjusted accordingly, and the calibration was repeated until the desired accuracy was achieved.

The system was controlled via an open loop control mechanism. The experimental protocol in terms of controlled variables Xc, LP and Qc was input into an Arduino code printer Python script, which outputs the corresponding Arduino code according to the calibrated model. The code was then uploaded to the Arduino microcontroller on the pump. Qc was set to a very low value ($Qc = 5 * 10^{-11} \frac{m^3}{s}$) in order to lower the shear stress on cells, which results in time delay of about 4-5 minutes before Xc reflects the changes in heights h1 and h2. This delay introduces a short time lag before changes in input Xc are detected in the cell culture region of the device. Greater flow rate can reduce this delay at the expense of higher shear stress on cells. In contrast with Xc, control over LP through h3 responds in under 10 seconds and is used to generate sharp pulses.

Experimental setup

Autoclaved Tygon tubes (Fisher Scientific 1471139) were washed with ethanol and PBS before multiple washes with DMEM medium. Tubes were inserted in the outlets of basins and media was again flushed through the tubes while carefully avoiding bubbles. Alexa647-conjugated BSA (0.0025 v/v; Invitrogen) solution in DMEM was used to prepare the TNF treatment which would later be used to quantify TNF concentration. For calibration experiments, mixtures of Alexa488-conjugated BSA and FluoSpheres (580/605, 2μm, Invitrogen) were used. This solution was flushed through the corresponding basin and attached tube. Basins with media and TNF treatment were placed on corresponding platforms on the pump with attached Tygon tubes. The other ends of the tubes were inserted in the corresponding inlets on the device. The tubes were clamped with plastic pinch clamps immediately after connection to the device to

avoid pre-treatment. The device was then fitted to a custom adapter and placed under the microscope for imaging. The clamps were removed at the beginning of the experiment. In experiments with translational inhibition, CHX (640 ng/mL, Sigma) and pan-caspase inhibitor q-VD-OPH (5µM, Thermo Fisher) were introduced to the dynamic stimulation device approximately 30 minutes before step and ramp stimulation.

Cell lines and culture

Hela cells stably expressing of EGFP-RelA as described previously (Lee et al., 2014) were cultured in Dulbecco's Modified Eagle Medium (DMEM) supplemented with 10% Fetal Bovine Serum (Corning), 100 µg/ml Streptomycin and 100 U/ml Penicillin (Corning, 30-002-Cl), and 0.2 mM L-glutamine (Corning, 25-005-Cl) at 37 °C and 5% CO2.

Microscope and live-cell imaging

Cells in the dynamic stimulation devices were imaged at 20X (0.45NA; Olympus) resolution with the climate controlled (37 °C, 5% CO_2) DeltaVision Elite microscope (GE). Constant flow rate of Qc = $5x10^{-11}$ m^3/s was used for all experiments as the model input. Cells continue to grow and divide in the device at these conditions for more than 12 hours. Time-lapse images were collected at 5-minute intervals using FITC and Cy5 filters.

Data analysis and trajectory classification

Trajectories of nuclear mean fluorescence intensity (MFI) in both EGFP-RelA (FITC channel) and Alexa647-conjugated BSA (Cy5 channel) were collected with custom ImageJ scripts. The EGFP-RelA trajectories were normalized to the average nuclear MFI of the three frames prior to the introduction of TNF to establish the baseline for fold-change transformed trajectories. Alexa 647 BSA trajectories were normalized with the maximum intensity (final intensity in case

of ramps) to get the fractions intensity at each time point. These normalized trajectories were then used as a proxy for volume fraction (Xc) to determine TNF concentration for each cell as $C = C_{max}$*Xc. Every trajectory was smoothened by a 3-frame moving average to remove high-frequency noise. Cells undergoing division, cell death, or that left the viewing area over the duration of the experiment were excluded from analysis. In addition, for experiments with CHX cells that underwent significant morphological changes or changes in overall expression of EGFP-RelA were not considered further.

The EGFP-RelA fold change trajectories were sorted into four categories: Adaptive (A), Sustained (S), Increasing (I), and Non-responding (NR) using the automated feature-extraction and classification algorithm defined in Figure S3. Four properties of nuclear EGFP-RelA fold change were extracted for each single cell trajectory and used to classify the type of response. A nuclear FP-RelA fold change threshold (f_{thresh}) value of $f_{thresh} = 1.4$ was used with maximum nuclear fold change over the entire time course (f_{maxTot}) to classify 'NR' and 'A' response modes. Remaining cells in the responding category were further classified as 'S' or 'I' by relating their nuclear EGFP-RelA fold change over the final 60 minutes of the trajectory (f_{final}) to f_{thresh} and the maximum fold change over the first 150 minutes of the trajectory (f_{max150}) during which time adaptive responses have already achieved maximal nuclear EGFP-RelA. Simulated trajectories were classified in the same way, with the exception of the value for f_{thresh} which was set to 1.3 to account for the reduced amplitude of simulated responses to ramp stimulation. Conditions with CHX were also analyzed with the same f_{thresh} as simulated trajectories to account for the diminished response amplitude associated with the immunosuppressive effects of Q-VD-OPH, used to minimize cell death during these experiments. The classifications algorithm is exhaustive, that is every single-cell trajectory is classified into one of the four categories, and completely unsupervised.

AUC of nuclear FP-RelA calculation

The area under the curve for nuclear FP-RelA was calculated as described previously (Zhang et al., 2017). Here, the area bounded positively by the fold change of nuclear FP-RelA (FC) and the initial FP-RelA value of 1 after the time of stimulus i.e. defined by $\sum_{i=time\ stimulus\ begins}^{i=end\ of\ experiment}(\max(1, FC(i)) - 1)$.

Permutation test

For permutation tests, the AUC of FP-RelA from cells exposed to step and ramp stimulation conditions were combined and randomly distributed into 'Permuted step' and 'Permuted ramp' bins without replacement, preserving the size of the original step and ramp data sets. 10^6 permutations were performed and the difference between the means of 'Permuted step' and 'Permuted ramp' data was calculated to generate a histogram. Two-tailed p-values were determined by computing the fraction of permuted data sets where $\Delta\text{mean}_{\text{permuted}} \geq \Delta\text{mean}_{unpermuted}$ (Figure S8).

Simulated single-cell trajectories

The "Deterministic 2-Feedback with Competition" (D2FC) model (Lee et al., 2014) was used to investigate the role of negative feedbacks in producing the novel response modes. All parameters were used at default as described previously except for IKK activation (ka), used as proxy for TNF concentration in the D2FC, in addition to IkBα and A20 translation rates (ca and c1a, respectively). to simulate ramp stimuli, we modified the D2FC model with variable IKK activation rate defined as:

$$\frac{dKa}{dt} = \frac{Ka\ corresponding\ to\ continuous\ stimulus}{Total\ time\ (\text{sec})}$$

Therefore, for continuous stimulation, Ka is set to a constant value (Ka = 0.00025) at time t=0,

and for ramp stimulation, Ka increases linearly from 0 up to its final value (Ka_{final} = 0.00025) over the duration of simulation.

2D parameter sweeps for IkBα and A20 translation rates (parameters ca and c1a) were performed across two orders of magnitude around their default D2FC values using latin-hypercube sampling. The following region was used to model cell-to-cell variability around the default parameterization (red box, Figure 6B): IkBa translation rate in the range of 0.158-15.84(s^{-1}) and A20 translation rate in range 0.1-10(s^{-1}). To account for effect of cycloheximide, a similar region (gray box, Figure 6B) with lower values for both feedback parameters contained IkBa translation rates in range of 0.0316-3.16(s^{-1}) and A20 translation rates in range of 0.01-1(s^{-1}). For each parameter combination responses to step and ramp stimulation were simulated. Each trajectory was classified as Adaptive, Sustained, or Increasing as described for the live-cell data and used to define the parameter response space (Figure 6B). Because the simulations are deterministic and cells are exposed to the same TNF step or ramp, simulations did not produce any non-responsive trajectories. All single cell simulations were performed in MATLAB (MathWorks) using the ode45 solver.

Data and Software Availability

The datasets generated and analyzed in this study are available from the lead author on reasonable request.

SUPPLEMENTAL REFERENCES:

Harris, J., Lee, H., Vahidi, B., Tu, C., Cribbs, D., Cotman, C., and Jeon, N.L. (2007). Non-plasma bonding of PDMS for inexpensive fabrication of microfluidic devices. J Vis Exp, 410.

Lee, R.E., Walker, S.R., Savery, K., Frank, D.A., and Gaudet, S. (2014). Fold change of nuclear NF-kappaB determines TNF-induced transcription in single cells. Mol Cell *53*, 867-879.

Zhang, Q., Gupta, S., Schipper, D.L., Kowalczyk, G.J., Mancini, A.E., Faeder, J.R., and Lee, R.E.C. (2017). NF-kappaB Dynamics Discriminate between TNF Doses in Single Cells. Cell Syst *5*, 638-645 e635.
