# Supplementary material for: A System for Analog Control of Cell Culture Dynamics to Reveal Capabilities of Signaling Networks
Source: iScience. 2019 Aug 8;19:586–96. doi: 10.1016/j.isci.2019.08.010 (PMC6713801; doi:10.1016/j.isci.2019.08.010)
Supplement: Data S3. D2FC Computational Model, Related to Figure 6 — MATLAB files for the D2FC model of the NF-κB are provided. [file mmc7.zip › SupplementaryFiles_S3/D2FCModel_2014/Read_me.pdf]

Matlab simulation package for the Deterministic 2-feedback with competition (D2FC) model of NF-κB-driven transcription (Lee et al. 2014, *Molecular Cell*, 10.1016/j.molcel.2014.01.026)

Fold Change of Nuclear NF-κB Determines TNF-Induced Transcription in Single Cells

Robin E.C. Lee, Sarah R. Walker, Kate Savery, David A. Frank, and Suzanne Gaudet

Correspondence to Suzanne Gaudet: Suzanne_Gaudet@DFCI.harvard.edu

The included Matlab files were based on the D2F model of the NF-κB pathway described in Ashall et al. 2009, *Science*, DOI: 10.1126/science.1164860. These files include: simulation.m, nfkbmodel.m, parameters.m and processSim.m.
